# *Mycobacterium tuberculosis*: a new hitchhiker in the etiopathogenesis of periodontitis

**DOI:** 10.1097/JS9.0000000000001122

**Published:** 2024-01-17

**Authors:** ArunSundar MohanaSundaram, Namra Vinay Gohil, Maudlyn O. Etekochay, Premalkumar Patel, Swathi Gurajala, Shanmugarajan Thukani Sathanantham, Mugisha Nsengiyumva, Santosh Kumar, Talha Bin Emran

**Affiliations:** a School of Pharmacy, Sathyabama Institute of Science and Technology, Chennai, Tamilnadu; b Medical College Baroda and SSG Hospital Vadodara, Gujarat; c School of Pharmaceutical Sciences, Vels Institute of Science, Technology and Advanced Studies (VISTAS), Chennai, Tamilnadu; d Karnavati School of Dentistry Karnavati University Gandhinagar Gujarat, India; e College of Applied Medical Sciences in Jubail, Imam Abdulrahman bin Faisal University, Saudi Arabia; f Winterset Edge, Ingham Road, Owings Mills, Maryland; g Mount Sinai Medical Center, Alton Road, Miami Beach, Florida, USA; h Public Health and Scientific Research Center, Kigali, Rwanda; i Department of Pharmacy, BGC Trust University Bangladesh, Chittagong, Bangladesh; j Department of Pharmacy, Faculty of Allied Health Sciences, Daffodil International University, Dhaka, Bangladesh

**Keywords:** extrapulmonary infection, inflammation, mycobacterium tuberculosis, periodontitis

## Abstract

Periodontitis, a chronic inflammatory disease of the gums affects both the ligament and alveolar bone. A severe form of periodontal disease affects a strikingly high number of one billion adults globally. The disease permutes both the soft and hard tissues of the oral cavity leading to localized and systemic diseases. Periodontitis has a deleterious impact on systemic health causing diabetes, cardiovascular diseases (CVD), and other disease. The cause of the enhanced inflammatory process is due to dysbiosis and an unregulated immune response. Innate immune response and T cells trigger uninhibited cytokine release causing an unwarranted inflammatory response. The RANK- RANKL interaction between osteoblasts, immune cells, and progenitor osteoclasts results in the maturation of osteoclasts, which promote bone resorption. It is well established that dysbiosis of the oral cavity has been implicated in periodontitis. But emerging reports suggest that the pulmonary pathogen, *Mycobacterium tuberculosis* (Mtb), causes extrapulmonary diseases such as periodontitis. Many clinical case reports advocate the involvement of Mtb in periodontitis, which poses a threat with the surge of tuberculosis in HIV and other immunocompromised individuals. Fostering a better understanding of the mechanism, causative agents and control on inflammatory response is imperative in the prevention and treatment of periodontitis.

## Introduction

Highlights

- Periodontitis, a chronic inflammatory disease of the gums affects both the ligament and alveolar bone, consequently leading to localized and systemic diseases.
- A severe form of periodontal disease affects a strikingly high number of one billion adults globally.
- *Mycobacterium tuberculosis* (Mtb) may cause periodontitis, as an extrapulmonary manifestation. Reported clinical evidences advocate that the involvement of Mtb in periodontitis poses a serious public health threat with the surge of tuberculosis in HIV and other immunocompromised individuals.

**Figure 1:**
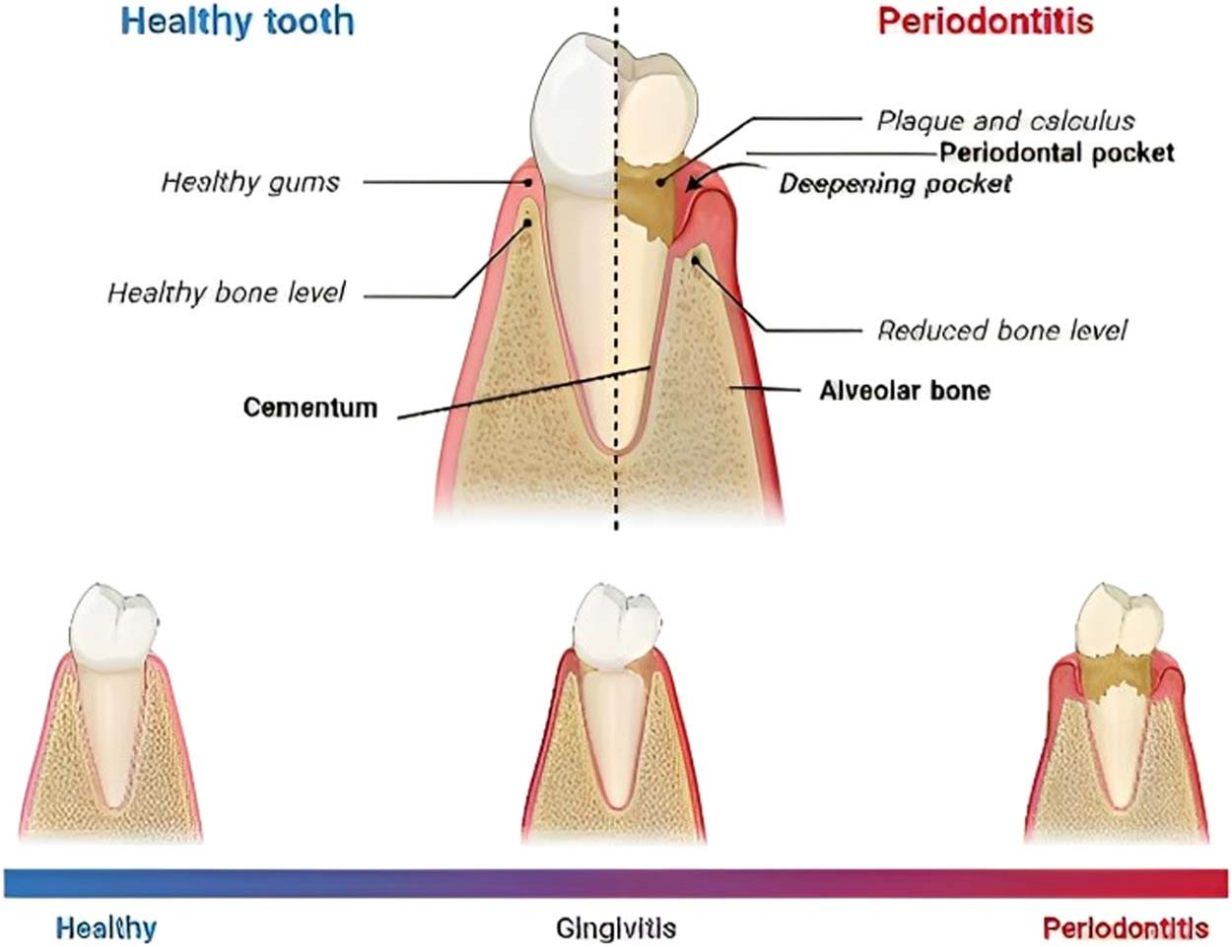
Pathological changes in the periodontitis.

Periodontitis is a chronic inflammation of the gums affecting the intact supporting tissues of the teeth^1,2^. The inflammation begins with bacterial biofilm in the oral cavity, which translates into clinical symptoms that includes erythema, bad breath, bleeding in the gums, tooth instability, and loss of tooth^3^. The pathology initiates from the loss of collagen, followed by bone destruction and resorption. In the early stages of periodontitis, patients do not experience discomfort due to a mild inflammation of the gums known as gingivitis (Fig. 1). However, when the disease reaches its severity, an enhanced inflammatory process leads to periodontal absorption culminating in tooth loss. In the last few decades, periodontal disease is a major threat to tooth loss, more than dental caries. Additionally, periodontitis exhibits several extraoral comorbidities such as heart disease, diabetes mellitus, nonalcoholic fatty liver disease, cancers, rheumatoid arthritis, and other diseases^4^. The pathology of periodontitis is determined by the disturbance of the oral microbiota and the corresponding elevated immune response toward the pathogens in the oral cavity.

This review comprehensively compiles the epidemiology, pathology, and comorbidities of periodontal disease. The article discusses the role of the pulmonary pathogen, *Mycobacterium tuberculosis* in the initiation of periodontitis.

Tuberculosis is a debilitating pulmonary disease transmitted by *M. tuberculosis*, an acid-fast bacillus. The bacteria spread from TB patients through aerosols to healthy people. On entry into the new host, pulmonary Mycobacterium resides in the lungs evading the host immune system by their ability to multiply within the host macrophages^5^. The host initiates a cell-mediated immune response that recruits lymphocytes to restrict the spread of infection. The alveolar macrophages release chemokines that attract the T-lymphocytes which along with other immune cells form granulomas, a cheesy hyper-immune region. Inside the granuloma, *M. tuberculosis* still survives as a nonreplicating bacillus. However, the pathogen powers the T-cell to induce a chronic inflammatory response resulting in necrotizing granulomas that spill the immune exudate and bacteria into the bronchial cavities to form aerosols^6^. The pathogen with its ability to resist the host immune system, especially in immunocompromised individuals rants into several comorbidities including COPD, lung cancer, diabetes, and obesity. Additionally, diagnosis of tuberculosis is limited by the long incubation of the pathogen without clinical symptoms, and poor detection. Recent advances implicate the dissemination of the pulmonary pathogen in oral periodontitis.

### Epidemiology

According to the WHO, a staggering one billion global adult population are suffering from periodontal disease, which is more prevalent in countries with low socio- economic conditions^7^. An epidemiological study from 1990 to 2010 showed about 58% increase in global periodontal disease with a cost burden of US $54 billion per year, and this trend has only increased in the last decade with increased life expectancy^8,9^. According to the Center for Disease Control^10^ about 47% of people above 30 years, and about 70% of people over 65 years suffer from periodontal disease. As per the same report, of the total 42% dentate population, 7.8% suffered from severe periodontitis and 34.4% from nonsevere form. Epidemiological studies of the three decades from 1990 to 2020 shows highest prevalence of periodontal disease in South-East Asia and Western Pacific followed by the Americas, Europe, and Eastern Mediterranean^11,12^.

Periodontal disease carries a multifactorial etiology with the major contributors being oral hygiene, smoking, and diabetes. Smoking increases the incidence of periodontal disease with smokers likely to suffer from the oral disease 2–7 times more than nonsmokers^13–15^. Periodontitis and many of the systemic diseases share a bidirectional association. Periodontitis promotes diabetes in many individuals, and in turn, people with chronic diabetes develop calculus on teeth leading to periodontal pocket at a greater frequency. The comorbidities associated with this oral pathology makes periodontitis, a major challenge^16^. In spite of being easily preventable, periodontal diseases are wide spread among the global population. The prime reason for tooth loss and threats to oral health is poor or limited oral treatment.

Tuberculosis is one of the most dominant epidemics in the South-East Asian and African regions. The countries most affected by the TB burden are India, Indonesia, China, and the Philippines. Between 2000 and 2021, ~2 million succumbed to tuberculosis. However, the co-relation between tuberculosis and periodontitis is in the nascent stage and thus poorly explored except for documented case studies. Chowdhary *et al*.^17^, reported the history of periodontitis in 700 patients suffering from pulmonary disease including tuberculosis. The study reported that 266 of the total 700 diagnosed with tuberculosis also showed periodontitis. There are very few studies on the clinical relevance of tuberculosis on periodontal health. Both tuberculosis and periodontitis evoke prolonged inflammation and is the cause of the clinical symptoms. This suggests periodontitis is a sequela of tuberculosis and hence it is critical to investigate the incidence of periodontitis in tuberculosis patients. However, the duration of tuberculosis and the extent of the disease has a correlation on the severity of periodontitis. It is probably vital for dental examination in TB patients.

### Oral microbiome and dysbiosis

The pathophysiology of periodontitis is determined by resident microbial species and the corresponding chronic immune reaction by the host toward the effect of the bacterial population^18^. The oral cavity is crowded with ~800 different types of bacteria making it the second-largest repertoire of microbiota after the gut. Hard and soft palates, tongue, lateral surface of the tooth along the gingiva are distinct niches for bacterial enrichment^19^. The papillae on the tongue support anaerobic life and therefore have a diverse microflora while the buccal and palatal mucosa shows poor microbial diversity. The oral cavity with its ideal temperature, pH, and lubrication by the saliva makes it a prominent cubbyhole for a healthy microbiota. The bacterial flora once established maintains the ‘microbial homeostasis’ to promote commensalism and mutualism^20^. The host gives an environment for the microbial communities to proliferate, and the commensal in return, block adherence, and proliferation of pathogenic bacteria^21^.

The oral microbiota plays a major role in health and its imbalance results in disease. It is mandate to understand the different species and their role in host-pathogen interactions. The Keystone Plaque Hypothesis (KPH) states that that certain microbial species, even in low numbers can trigger inflammation by increasing the normal microbiota (Hajishengallis et al., 2012). The bacterial species are predominantly from the phyla, Tenericutes, Spirochetes, Proteobacteria, Firmicutes, Fusobacteria, Euryarchaeots, Chlamydia, Bacteriodetes, and Actinobacteria^22^. *Porphyromonas gingivalis* is one of the *keystone pathogens*, which promotes periodontitis by causing dysbiosis^23^. A current addition is the candidate phyla radiation, which comprises about a quarter of the Earth’s bacterial diversity from 73 different phyla but yet placed in a single phylum. Members of the candidate phyla radiation Saccharibacteria phylum are ubiquitous in the human oral microbiome and hence can influence the oral health and their imbalance can cause diseases such as periodontitis^24,25^.

The oral niche is challenging for microbes because of the temperature, pH, shear force, nutrients and hence the microbiota has evolved strategies to survive in the oscillating environment. Additionally, life style such as dental hygiene, smoking habits determine the biotic population. The bacteria in the oral cavity exists in the form of biofilm to overcome the fluctuating environment of the oral cavity. The next-generation sequencing has made it possible to document the 800 different bacterial species into 185 different genera belonging to 12 different phyla. The 16S rRNA was sequenced and the Human Oral Microbiome Database was developed, and the different bacteria species of microbiota can be accessed (www.homd.org)^26^. Table 1 is a representative of species of bacteria belonging to different phyla.

**Table 1:** Distribution of different taxa.

Phyla	Total number of species	Examples of species
*Firmicutes*	227	*Streptococcus infantis, S.oralis, S.peroris, Enterococcus fecalis, Lactococcus lactis, S.sanguinis. L. casei, Gemella sanguinis*
*Fusobacteria*	32	*Fusobacterium nucleatum, F. periodonticum, Sneathia sanguinegens, Leptotrichia goodfellowii, L. buccalis*,
*Proteobacteria*	106	*Burkholderia cepacia, Kingella denitrificans, Simonsiella muelleri, Kingella oralis, Neisseria mucosa*
*Actinobacteria*	72	*Actinomyces radicidentis, A. odontolyticus, Rothia dentiocariosa, R. mucilaginosa, Propionibacterium acidifaciens, Corynbacteria diphtherium*
*Bacteroidetes*	107	*Prevotella denticola, P. P. multiformis, Bacteroides zoogleoformans, Tannerella forsythia, Porphyromonas gingivalis*
*Chlamydiae*	1	*Chlamydophila pneumoniae*
*Chloroflexi*	1	*Chloroflexi [G-1]* sp.
*Spirochetes*	49	*Treponema vincentii, T. denticola, T. pallidum*
SR1	1	*SR1 [G-1]* sp.
*Synergistetes*	10	*Jonquetella anthropic, Pyramidobacter piscolens*
*Saccharibacteria* (TM7)	1	Uncultivated phenotype
*Gracilibacteria* (GN02)	1	Uncultivated phenotype

**Figure 2:**
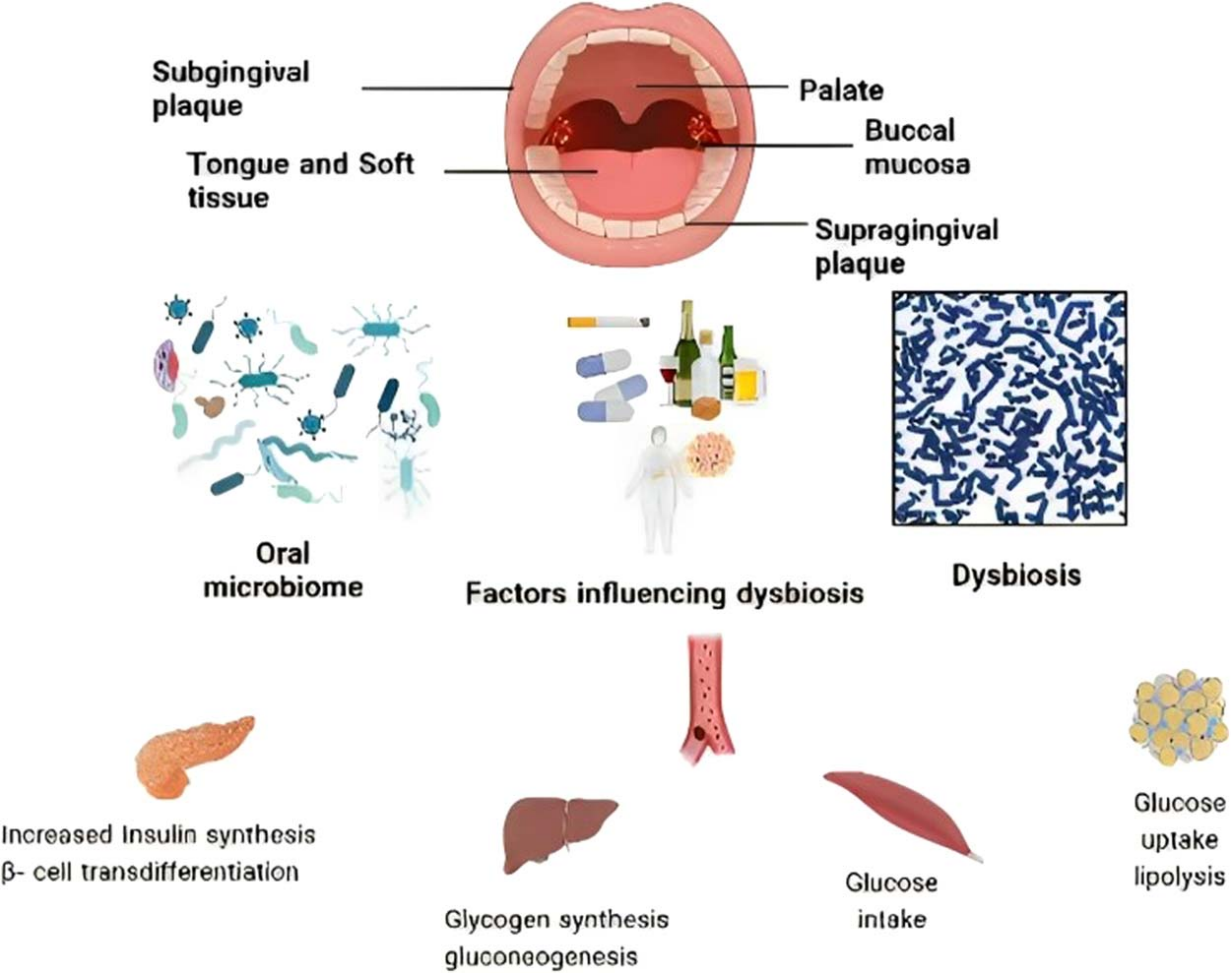
Dysbiosis in periodontitis and the influencing factors.

Dysbiosis is an alteration of the diversity and relative abundance of the various species of the resident microbiota^27^. Periodontitis occurs due to a profound shift in the constitution of the subgingival bacterial population, with pathogenic gram‐negative species outgrowing health‐associated taxa. The population of the pathogens increase in the oral cavity, which promotes chronic inflammation. The pathology of periodontits is not determined by a single bacterial species but by the dysbiosis of the oral microbiome (Fig. 2). Keystone pathogens remodel the oral microbiota from commensal to dysbiotic by disruption of host homeostasis^28^.

The keystone pathogens activate the host immune response remodeling the oral microbiota into dysbiosis, which initiates tissue destruction. Among the enriched species are the classically described red‐complex triad - *Tannerella forsythia, Porphyromonas gingivalis*, and *Treponema denticola* are the major causative agent of the periodontits^29^. Several studies specify a high concentration of spirochetes in periodontal destruction. Healthy communities develop gingivitis when the relative abundance of *Rothia dentocariosa*, *Propionibacterium*, and *Stenotrophomonas maltophila* decreases while the population of *Prevotella* spp. and *Selenomonas* increases *in plaques* ^30^. Certain pathogenic species of Actinobacteria, Candida, and Fusobacterium become resident in the oral cavity promoting different pathologies of periodontitis. Dysbiosis triggers host inflammatory response in gingival crevicular fluid thereby increasing mediators such as IL-1, and its receptor IL-1Rα, TNF. The inflammatory response promotes comorbidities such as diabetes. This is achieved through increased circulating glucose by gluconeogenesis and free fatty acids through lipolysis^31^.

### Oral lesions in periodontitis

The periodontium is comprised four major components: alveolar bone, periodontal ligament, gingiva, and cementum^1^. Peridontitis, an inflammatory disease of the gums includes gingivitis and periodonotitis. The cascading gum abrasion event is a consequence of both unrestrained inflammatory response and bacterial colonization on the surface of the teeth and gums^32,33^. The chronic inflammatory response to pathogenic invasion extends to the destruction of the periodontium^34^. The pathology of gum diseases begins with mild gingivitis to chronic gingivitis leading to inflammation of deeper tissue causing periodontitis^35^.

The periodontal disease can be stacked at three levels: initiation, exacerbation, and resolution of the disease. The chronic oral disease initiates with a small-grade inflammation observed as neutrophil recruitment in the gingiva with no obvious evidence of pain or inflammation. The microbial biofilm or plaque is usually formed on the margin of the gingiva. When the inflammation fails to clear the biofilm it leads to gingivitis, an inflammation of the gums, which in some cases translate into periodontitis. Gingivitis is a superficial gum disease while periodontitis is an irreversible damage, which affects the periodontal ligament and alveolar bone. There are no single bacteria but dysbiosis of the oral microbiome contributes to the metamorphosis from chronic gingivitis to cataclysmic periodontitis.

Exacerbation switches the pathology from gingivitis to periodontitis, which coincides with an altered subgingival microenvironment and a corresponding chronic inflammation. The immune response can contribute to destruction or repartition according to different circumstances. Both dysbiosis and chronic inflammation are the drivers of periodontal disease. A reciprocal relationship between the inflammatory response and dysbiosis promotes tissue disintegration. Dysbiosis in the subgingiva drives further inflammation perpetuating disease. The clinical treatment is either reducing the inflammation or dysbiosis. This has been confirmed in animal studies where induced periodontitis has been treated with anti-inflammatory drugs or reversed dysbiosis (Lee *et al*., 2016, Eskan *et al*., 2012). The pathogens of the periodontum activate both innate and acquired immune response, which exacerbates the inflammation and disease. Chronic inflammation causes irreversible damage to both soft and hard tissues of the periodontium. Prolonged chronic inflammation proceeds to systemic inflammation extending the oral disease to other tissue initiating several nonoral diseases.

Periodontitis results from a failed resolution of inflammation, which may be due to a delay in neutrophil apoptosis, inability of regulatory T cells to eliminate inflammation. Lymphangiogenesis facilitates the clearance of inflammation and its delay or failure retards the resolution of periodontitis.

Periodontal diseases can be defined on the basis of inflammation and tissue damage.

1. Acute periodontal diseases set in rapid and are associated with mild pain or discomfort, tissue destruction, and infection. They include abscess in gingiva and periodontium and if untreated can lead to rapid destruction of the tooth tissues and may also contribute to systemic diseases.
2. Aggressive periodontitis involves quick attachment loss of tooth and destruction of bone, and occurs suddenly in clinically healthy individuals.
3. Chronic periodontitis, a most frequent form of periodontitis begins with gingival pocket formation leading to the destruction of alveolar bone and tooth loss.
4. Systemic periodontitis: shows symptoms from a young age and occurs in patients with systemic diseases such as diabetes, and heart disease.
5. Necrotizing periodontal diseases is an aggressive oral disease that marks both soft tissue (gums) and hard tissue (alveolar bone).

### Periodontitis and comorbidities

Reports clearly demonstrate that chronic periodontitis can upgrade to low-level systemic inflammation, which can translate into several comorbidities^36^. Proinflammatory cytokines such as IL-1, IL-6, C-reactive proteins, which cause vascular damage, and activated neutrophils are high in patients with chronic periodontitis, which trigger systemic response. Several reports indicate a strong correlation between periodontitis and other systemic diseases such as cancer, diabetes mellitus type-1, and rheumatic diseases. A study of 11 869 individuals showed an association between oral and systemic inflammation in CVD^37,38^. In another study of 10 877 people who received a dental treatment showed decrease incidence of acute myocardial infarction and stroke compared to the control group which did not receive dental check-up^39^. Systemic inflammation from periodontitis is caused by the dissemination of bacteria or inflammatory molecules from the oral tissues to the bloodstream. The ulcerated periodontal pockets ranging in size from 10 to 20 cm^2^ drain the bacteria and their antigens into circulation. Inflammation at the extraoral sites such oropharyngeal or orodigestive causes pulmonary and gut mediated spread of systemic inflammation.

**Figure 3:**
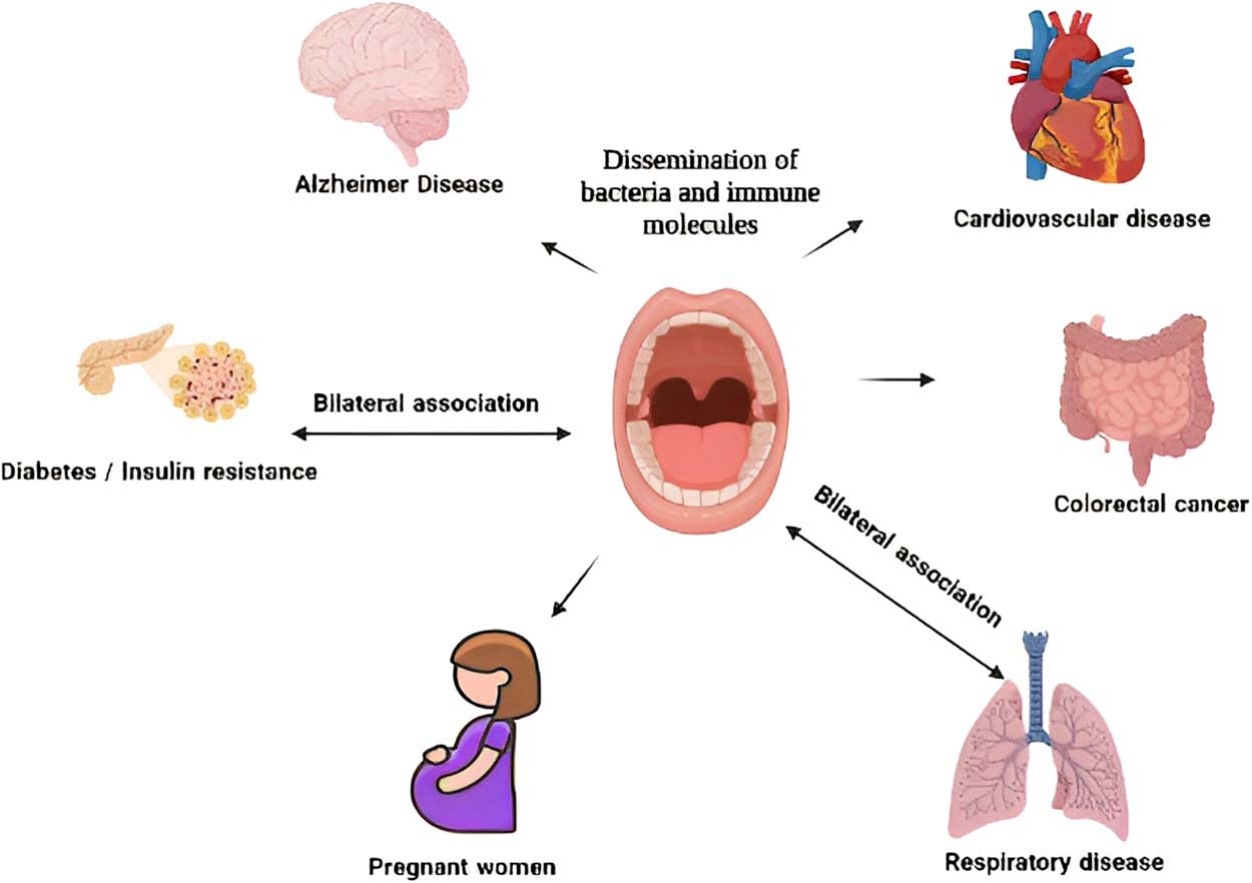
Systematic complications of periodontitis.

From the periodontal pockets, the microbes reach different regions of the body via gingival crevicular fluid. Enhancer proinflammatory mediators such as TNF-α, IL-1, IL-6, IL-23, and IL-17 result in the destruction of synovium. Periodontal pathogens may cross the brain-blood barrier, enhancing inflammation in the brain to drive neuropathology in diseases such as Alzheimer’s disease^40^ (Table 2). Offenbacher *et al*.^41^ first reported the association of periodontitis and low birth weight in a case–control study of 124 patients. A recent review accentuated that severe periodontitis can lead to premature low birth weight, and many other birth complications. Obstetric periodontal pathogens stimulate chronic inflammatory responses in the placenta leading to elevated proinflammatory cytokine in the fetal tissues. *Fusobacterium nucleatuem* and *Porphromonas gingivalis* are largely implicated in preterm birth defects because of their ability to induce placental inflammation leading to adverse reactions^42^. Periodontitis increases systemic inflammatory burden causing several metabolic disorders, such as obesity, T2DM, nonalcoholic fatty liver diseases, and CVDs (Fig. 3). Elevated levels of low-density lipoprotein and triglycerides are observed in patients with periodontitis and treatment of the gum disease reduces the lipid levels. Abnormal biochemical markers are observed in periodontitis induced in experimental animals. The association between diabetes and periodontitis is bidirectional as diabetes enhances the pathology of periodontitis, and in inverse, periodontitis seem to contribute to diabetes in patient with poor oral health^43^. The pathogenic population of periodontitis causes metabolic defects such as increased insulin resistance and lipidemia. Circulating glucose is enhanced by increasing hepatic glucose levels through FOXO1 mediated gluconeogenesis^44^. Periodontitis is associated with increased oxidative stress achieved by inflammatory responses. This results in abnormal levels of adipokines, a major factor that contributes to increased lipolysis^31^.

**Table 2:** Comorbidities of periodontal diseases.

Disease	Pathology
Cancer	Increased TNF-α, IL-1, and IL-6 are observed in periodontitis which may manifest into cancer
Diabetes	Increased levels of TNF-α correlates with diabetes
Obesity	Metabolic endotoxemia caused by dysbiosis increases the concentration of lipopolysaccharide which increases obesity through exacerbated TNF and IL-6 levels. These cytokines also contribute to onset of insulin resistance
Atherosclerosis	Proinflammatory cytokines such as TNF-_ and IL-6 lead to dysfunction o endothelium causing impaired vasodilation. This over a period of time creates atherosclerotic plaques which induce inflammation of the vascular tissue
CVD	Chronic periodontitis promotes bacteremia leading to systemic inflammation. The cytokines trigger endothelial lesion which translates into platelet aggregation causing chronic vascular diseases
Respiratory diseases	The bacteria inversion from periodontal pockets to systemic circulation via gingival crevicular fluid triggers cytokine storm causing pulmonary congestion
Rheumatoid arthritis	*P. gingivalis* triggers secretion of autoantibodies causing rheumatoid arthritis. Abnormal proinflammatory mediators results in destruction of the synovium
Neurodegenerative diseases	Many of the dysbiotic bacteria can cross the blood brain and can cause brain inflammation. Some of the reported cases of Alzheimer’s disease is due to chronic periodontitis.

Diabetes promotes periodontal disease in many ways – dysbiosis of the oral microbiota, formation of advanced glycation end products (AGE) in poorly controlled diabetes and these modified glycation products increase proinflammmatory cytokines leading to enhanced bone resorption and finally tooth loss^45^. Periodontitis increases both proinflammatory and anti-inflammatory cytokines, which are soluble proteins that diffuse into the systemic circulation creating a perpetual inflammatory reaction^32,46^. Periodontitis seem to be related to induce autoimmune diseases such as rheumatoid arthritis and cancer such as colorectal neoplasm in patients.

Considering the receding dental health and the association of periodontitis with several comorbidities, there is a need for novel customized therapies that can treat both the diseases. Treatment of periodontitis on comorbidities were restricted primarily to diabetes. The most prominent treatment is antibacterial agents as adjuvant therapy^47^ reported administration of doxycycline hyclate in the sub-gingivitis in patients with type I diabetes. Another study by Das *et al*.^48^ confirmed that DM patients benefitted on doxycycline as adjuvant therapy over conventional antimicrobial treatment. Combined adjuvant therapy of metronidazole and amoxicillin along with scaling and root planning (SRP) in diabetic patients improved the pathogenesis^49^. The hypoglycemic drug, metformin was found to be effective in the treatment of periodontitis and diabetes^50^. Propolis has been used as an adjuvant with SRP in periodontitis that demotes fasting blood sugar levels^51^. Also, drugs that reduce inflammation seem to relieve patients suffering from periodontitis and diabetes.

### Immune mechanism in periodontitis

In healthy state, a balance exists between the oral microbiome and a mild host immune response. However, when the periodontium gets infected with keystone pathogens they hyperactivate the immune system causing tissue destruction. Porphyromonas gingivalis manipulates the innate immune system (reviewed by^28^) for its own survival, and also other pathogenic members of the bacterial community. The keystone pathogens have the ability to promote inflammation even when they are in relatively few numbers^52^.

Several mechanisms contribute to the pathology of periodontitis. A complex network of both innate and adaptive immunity is triggered during periodontal disease^53^ (Güler *et al*., 2020). The inflammatory cascade begins with activation of toll like receptors (TLRs) identifies infectious molecules, pathogen associated molecular patterns.^54^ TLR-2 recognizes the lipoproteins and peptidoglycan of subgingival bacteria, TLR-4 lipopolysaccharide of gram-negative bacteria. Activation of TLR signaling pathways are divergent, ranging from proliferation, differentiation and maturation of immune cells such as PMNs, macrophages, B cells, NK cells and T cells^55,56^.

**Figure 4:**
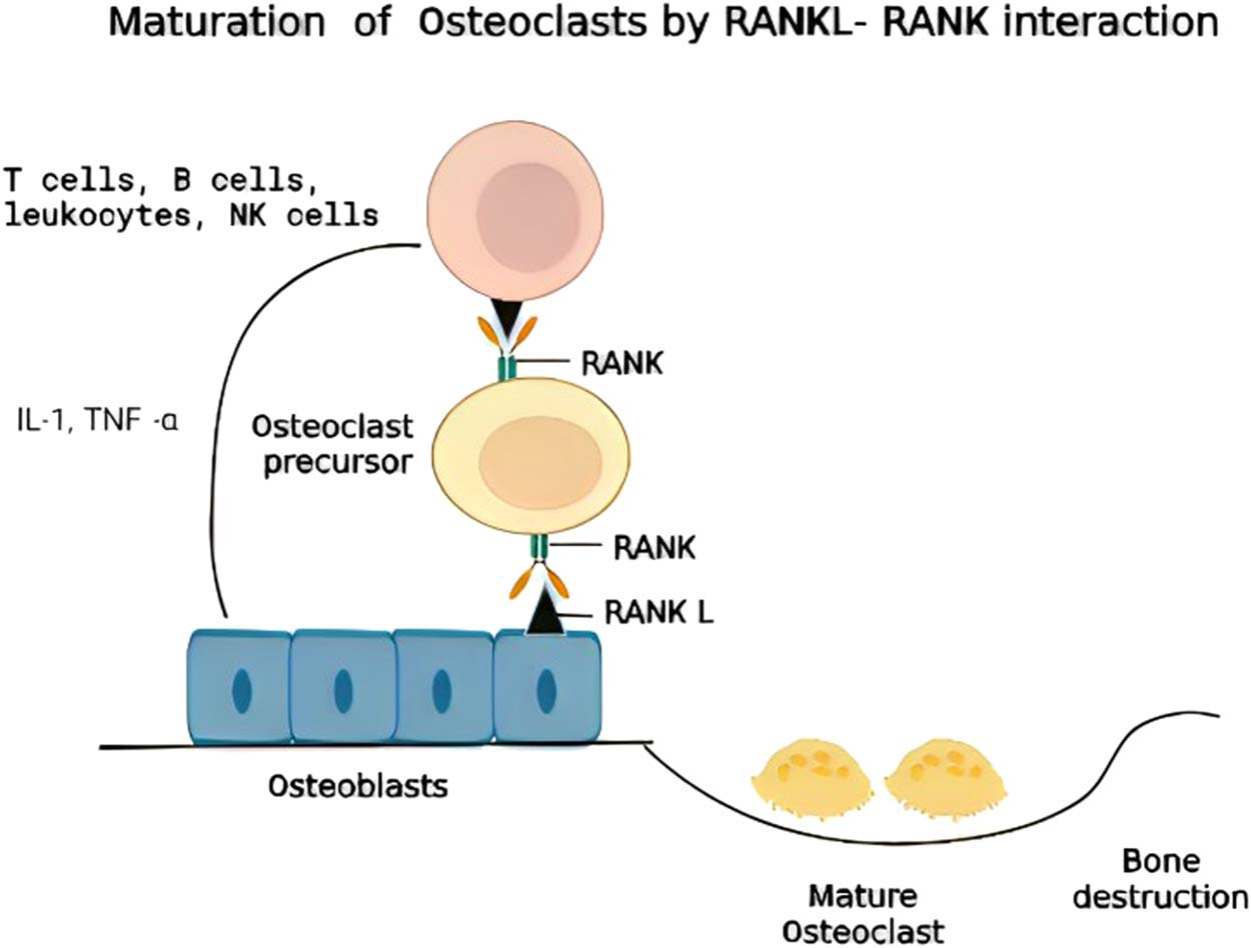
RANKL- RANK interaction promotes maturation of osteoclast which is involved in alveolar bone destruction.

Lipopolysaccharide of gram-negative bacteria stimulates macrophages to release TNF-α, IL-1β, and prostaglandin E2 (PGE2). These factors promote release of matrix metalloproteinases from macrophages, epithelial cells, and fibroblast, which remodel extracellular proteins such as collagen fibers causing severe damage to periodontium. The proinflammatory cytokines increase expression of nuclear factor kB ligand (RANKL) in osteoblasts and T-helper, which interacts with its cognate receptor RANK on precursor osteoclast. The RANKL-RANK interaction promotes maturation of osteoclast, which is involved in alveolar bone destruction (Fig. 4)^57,58^.

Innate immune cells such as macrophages, dendritic cells, natural killer (NK) cells, B/T-lymphocytes of the adaptive immune response, and proinflammatory cytokines such as IFN-γ, IL-1, and IL-6 play a huge role in periodontal disease^59^. In the initial stages of periodontitis, the inflammatory response protects against bacterial infection, but dysregulated and prolonged inflammation results in destruction of periodontal tissues^46^. Chronic inflammation in the subgingival region causes proinflammatory molecules to stream into the systemic circulation and disrupt the systemic health^60,61^. It has been reported that relative to healthy subjects, activated immune cells and inflammatory molecules such as C-reactive proteins are relatively higher in people with periodontitis^62^. Certain cytokines differentiate CD4+ T cells into subpopulations of Th1, Th2, Th17, and Treg cells. Th1 and Treg carry anti-inflammatory properties in periodontitis while activated Th2 destroy B cells in chronic periodontitis^63^. Th17 cells activated by IL-17 and IL-23 are potent inducers of tissue inflammation. Under normal conditions, cytokines released by innate and adaptive immunity regulate tissue homeostasis. However, disruption of the balance during periodontitis results in chronic inflammation^64^.

The proinflammatory cytokines are IL-1α, IL-1β, IL-18, IL-33, IL-36, IL-37, and IL-38 (Table 3). During periodontitis, monocytes, and macrophages are recruited at the infectious site and releases IL-1β, which increases the blood flow thereby recruiting leukocyte and neutrophils, and activating both Th1 and Th2 cells, and stimulating bone resorption. These entire features make IL-1β play a central role in periodontitis. Pathogenesis of periodontitis is regulated by dysfunction of T cells resulting from excess inflammatory cytokines (Table 3). The cytokines modulate the local gum tissues by destruction of collagen fibers of gingiva, absorption of alveolar bone, and supporting tissue of the teeth, and finally tooth loss. In healthy individuals, the oral cavity is maintained by the immune surveillance of CD4+ T cells followed by smaller population of CD8+ T cells and γδ T cells, which helps in maintaining the dynamic balance between bone resorption and osteogenesis^65^. However, during invasion of periodontal tissue by pathogens, immune cells such as monocytes, macrophages, and dendritic cells are recruited leading to activation of T cells, and release of a dysregulated inflammatory cytokines, which activates osteoclastic activity leading to alveolar bone loss. The cytokine cascade has pleiotropic effect from recruitment of different immune cells, control of pathobionts, and induction or suppression of osteoclastic activity^66^.

**Table 3:** Cytokine network in Periodontitis.

Proinflammatory cytokine	Characteristic role
IL-1	Innate and adaptive immunity, enhances IL-2 levels by activating T-lymphocytes, increase immunoglobulin synthesis by B cells
IL-1α	Activates Th17indirectly by promoting the production ofIL-23 and IL-6
IL-1β	Bone resorption, activates both Th1 and Th2 cells
IL-2	Activation of Treg cells, NK cell; Differentiation of B and T cells
IL-6	Differentiation of CD4+ T cells via Th17, bone homeostasis through over expression of RANKL in osteoblasts
IL-12	IFN- production, plays protective role in in the pathogenesis of PD
TNF	Exacerbates bone resorption, connective tissue, attachment loss and up-regulates proinflammatory cytokines
IL-23	Secreted by myeloid APC on challenge with *P. gingivalis*, suppresses anti-inflammatory IL-10
IFN-γ	Major cytokine of Cell-mediated immune response, inhibits formation of osteoclast, controls periodontitis
TGF	Inhibits B cells, CD4 and CD8 T cells, IL-1 production, TNF-α, MMP, stimulates collagen synthesis

### Treatment of periodontitis

As periodontitis is an oral inflammatory disease, maintaining a good oral health is a mandate to prevent the disease. Researchers have been studying the different ways of delivering the drug to the target site as systemic delivery of antimicrobials has failed. As the bacteria form biofilms, it is extremely challenging to remove the pathogens from the site of inflammation. The first treatment involves brushing twice a day and flossing once a day to reduce the bacterial load. In patients who show mild to moderate symptoms, supragingival irrigation with 0.3% acetyl salicylic acid is recommended^67^. This treatment reduces both plaque formation and inflammation of the gums. Antiseptics such as Listerine and Peridex are recommended to avoid plaque formation and bad smell. In contrast, subgingival irrigation is relatively less efficient as it does not remove the plaque formation but can diminish the inflammation^68^. This can reduce the microbial numbers in the periodontitis pocket. SRP are better treatment procedures for periodontitis as it remove plaques and calculus from around the ridges of the teeth. SRP as a non operative treatment is the gold standard for periodontal pathogenesis. The treatment skillfully reduces the microbial count and controls bleeding in the gums. Ultrasonic SRP is capable of reducing the bacterial load to almost 0.1%, and recolonization of nonpathogenic strains. Host Modulation Therapy are gaining momentum in treatment of periodontitis^69^. These include use of doxycycline, bisphosphates, anti-inflammatory drugs to lower prostaglandins. Food and Drug Administration (FDA) has approved the use of tetracycline as an adjuvant drug to SRP. The latest treatment is the intrapocket drug delivery of antibiotics using membranes and films, which is a localized therapy with excellent prognosis^70^.

### Tuberculosis: the inter-relationship with oral health

Tuberculosis, a major illness of the lungs can also cause extrapulmonary diseases in the bones, spine, brain, intestines, and several other tissues. However, oral tuberculosis is infrequent with only 0.1–5% of total tuberculosis cases reported. Though *M. tuberculosis* in oral samples is almost universal in patients with pulmonary tuberculosis, in very rare cases they are resident in the oral cavity to cause disease^71^. The pathogen has been isolated from the plaque and saliva of patients with periodontitis as a secondary inoculum. But oral tuberculosis has been reported in both young and adults as an asymptomatic disease. The swabs of oral cavities from tuberculosis patient reveal *M. tuberculosis* and *M. bovis*. Generally, it is believed that oral lesions by Mycobacterium are considered a secondary response to pulmonary infection. The microbe from the sputum may enter the oral cavity through abrasions or by a haematogenous route during pulmonary infection^72^. The oral environment such as poor oral hygiene enhances the odds of oral tuberculosis. Individuals with pre-existing lesions such as leukoplakia, abscess cysts, gingivitis, and periodontitis are susceptible to oral tuberculosis. The pathological presentation of oral tuberculosis is predominantly ulcers with various symptoms. They can be either single or multiple deep, superficial or deep lesions accompanied with or without pain. It may begin with a opalescent vesicle around which caseation due to chronic inflammation to the microbe develops. Around the vesicle, tiny nodules called sentinel tubercles are observed. Hamid *et al*. reported four cases of primary oral tuberculosis bearing clinical pathology in gingiva, tongue, and palate. The cases were confirmed for tuberculosis by histopathological examination, PCR, and Mantoux test. Each of the cases showed different clinical symptoms – nonpainful gingival swelling, nonpainful gingiva ulcer, painful ulcers on the palate and tongue.

### Primary and secondary oral tuberculosis

Oral tuberculosis are of two types – primary and secondary infection. Primary infection is when the oral cavity is directly infected by the bacteria rather than dissemination from the lungs^73^. Lesions caused by primary infection are rare and reported in children and mostly in gingiva, mucobuccal fold, and regions where tooth have been extracted and leads to gingival enlargement. In contrast, secondary oral tuberculosis is more frequent in adults and is a consequence of auto inoculation of live mycobacteria from the sputum. The lesions are found in the regions of tongue, palate, lips, and jaw bones. Karthikeyan *et al*. reported two cases of oral primary tuberculosis translating to gingival enlargement. Lesions caused by tuberculosis are chronic, nonhealing, and irregular shaped ulcers. They appear diffused, papillary projection of the gingival tissue. The lesions appear as ulcers of the oral cavity with hardening of the soft tissues and tuberculosis osteomyelitis of the jaw has been reported (Dixit, 2006)^74^. The ulceration begins slowly with different morphologies from irregular, superficial, and deep lesions, which may be accompanied with pain and erythema and induration with yellowish granular base.

The oral tuberculosis lesions carry different pathological features such as nodules, fissures, plaques, vesicles, and granulomas and are found in the maxilla and mandible. Primary tuberculosis appears as fiery red, irregular, papillary proliferation with granular appearance on the gingiva. With HIV/Tuberculosis posing a major threat to global health, we will observe and unusually high increase in extrapulmonary tuberculosis such as oral tuberculosis. Hence, it is critical to understand the epidemiology, mechanism, and pathology to prevent misdiagnosis of oral tuberculosis.

### Clinical manifestation of oral tuberculosis

Acute miliary, chronic ulcerative, and lupus vulgaris are the different forms of oral TB^72^. Over 93% of all oral lesions are ulcers primarily restricted to the tongue^75^. Tuberculosis, a chronic systemic granulomatous disease has been reported to affect the oral cavity as a secondary inoculum. Primary infection or oral cavity by tuberculosis is yet to be reported but oral tuberculosis is the consequence of the spread of the disease from other regions of the body. Brody *et al*. (1922) and Kramer (1925) reported about 100 years ago the association between periodontitis and tuberculosis.

### Classification of orofacial tuberculosis

*Tuberculous ulcer can* be of various kinds – painful or painless, single or multiple, deep, or superficial. The typical lesions are stellate ulcer found on upper side of the tongue, gingiva, palate, lips, and buccal mucosa. The features of tuberculosis ulcer are irregular with thickening on the margin, necrosis at the base surrounded by tiny nodules called sentinel tubercles. Pathological features of oral TB include nodules, fissures, plaques, vesicles, tuberculomas, and granulomas^76^. The lesions of TB peridontitis are found more on the hard than soft palate. Tuberculous ulcer can be categorized as traumatic, syphilitic, and pathos ulcers and when untreated can transform into oral squamous cell carcinoma.
*Tuberculous gingivitis* affects only the gingiva as nodular and papillary and usually does not affect the alveolar bone or exhibit cervical lymphadenopathy. The clinical symptoms also include gingival enlargement, periodontitis, and loss of tooth and inflammation of cervical lymph nodes.
*Tuberculous dental periapical granuloma: M. tuberculosis* can infect the oral cavity through deep caries via saliva, hematogenous, and through periodontal pocket.
Tuberculous in post extraction of tooth: After extraction when the healing process gets delayed, the tooth socket gets filled tuberculous granuloma consisting of red elevations.
*Tuberculous lymphadenitis:* Tuberculous lymphadenitis is the most common extrapulmonary TB accounting for 5% of the cervical lymphadenopathy. It is observed as a mass in the neck affecting the lymph nodes. Patients develop slowly enlarging lymph nodes in the neck and are treated surgically, or chemotherapy.
*Lupus vulgaris:* It is cutaneous tuberculosis that spreads through blood or lymphatic route. The lesions are prominent on the neck, face, nose, eyelids, lips, and ears. It is more prominent in females compared to males. The skin lesions are of five types: plaque, ulceration, vegetating, tumorous, and papulonodular.

### Early evidence of *M. tuberculosis* induced periodontitis

Keystone pathogens and other microbes has been implicated in the establishment of periodontitis. A new emerging pathogen in periodontitis is the pulmonary pathogen, *M. tuberculosis*. The acid-fast bacilli causes extrapulmonary disease but was not implicated in the prognosis of periodontitis^77^. In the last few years, there are case reports on the involvement of Mtb in periodontitis. Hamid *et al*.^73^, reported four cases of primary oral tuberculosis. The resident pathogen was established by biopsy of the oral tissue, PCR, and Mantoux test. The patients did not show positive chest radiography and sputum test, which are the classical diagnostics for pulmonary tuberculosis. Histopathology revealed cluster of epithelial cells, caseous necrosis, and giant cells a classical feature of chronic inflammation associated with intracellular bacteria such as *M. tuberculosis*. A case report of a 37-year-old female described a presentation of swelling of the anterior gingiva without pain. The patient complained of loss of appetite and weight in the last 3 months without any other systemic repercussions, dental trauma, lymphadenopathy, or any surgery in the affected region. Oral examination showed enlargement of the labial maxillary gingiva with red, granular appearance, bleeding on pressure from 13 to 23 tooth. The patient was treated with antituberculosis drugs.

In another report from the same study, the patient showed palate ulcer symptoms that increased with time. Antibiotics such as amoxicillin did not improve the condition, which suggested that the pathogen was not gran negative. No systemic complaints such as fever, cough, and also no lymphoadenopathy. The ulcer was irregular with a necrotic base. It was suspected to be malignant and biopsy on the sample was performed. Histopathological reports showed caseation granulomas with no neoplastic transformation. The results suggested a possible oral TB and the Mantoux test was positive with no lesions in the chest radiography and sputum negative. It was diagnosed as TB due to caseation granulomas surrounded by lymphocytes, and giant cells. The patient was treated with isoniazid and ethambutanol and the patient showed improvement in 4 weeks^73^. Two such case reports established the involvement of tuberculosis in periodontitis.

Another report by Garg *et al*.^78^, shows orofacial tuberculosis.^79^ report the challenges associated with orofacial tuberculosis at different regions of the oral cavity including the gums. However, advanced techniques have made the identification of tubercle bacilli in periodontitis relatively easy and reliable. This is critical for it helps in early treatment of the disease without loss of alveolar bone.

## Conclusion

Oral health determines the overall health of an individual. Periodontitis, a chronic inflammatory gum disease is caused by dysbiosis of the oral cavity. Keystone pathogens aggravate the immune system leading to gum damage to form periodontal pocket. Periodontitis is a consequence of the tussle between the host immune system and the oral microbiota. Tubercle bacilli as a causative of periodontitis are an upcoming concern in oral health. The acid-fast bacilli have been identified in oral tissues due to easy access of sputum in the oral cavity and it is spread across the mouth and its role in disease. The role of tuberculosis in periodontitis is still not thoroughly established, emerging clinical investigations have proved the role of extrapulmonary tuberculosis in periodontitis. Further research and better clinical understanding and diagnosis is required for identification of the acid-fast bacilli in periodontitis.

## Ethical approval

Not applicable.

## Consent

No ethical approval required for this type of article.

## Sources of funding

This paper received no specific grant from any funding agency in the public, commercial, or not-for-profit sectors.

## Author contribution

A.M.: conceptualization, revision, and supervision; N.V.G., S.T.S., and M.O.E.: writing original draft; P.P. and S.G.: data collection and editing; M.N.: editing and revision; S.K.: critical review and revision; T.B.E.: data collection, revision, and supervision.

## Conflicts of interest disclosure

The authors declare no conflict of interest.

## Research registration unique identifying number (UIN)

1. Name of the registry: not applicable.
2. Unique identifying number or registration ID: not applicable.
3. Hyperlink to your specific registration (must be publicly accessible and will be checked): not applicable.

## Guarantor

Talha Bin Emran, Associate Professor, Department of Pharmacy, BGC Trust University Bangladesh, Chittagong 4381, Bangladesh. Tel.: +88 030 3356193, fax: +88 031 2550224, Cell: +88 01819942214. https://orcid.org/0000-0003-3188-2272.

ArunSundar MohanaSundaram, Associate Professor, School of Pharmacy, Sathyabama Institute of Science and Technology, Chennai, Tamilnadu, India. E-mail: arun.laureate@gmail.com; arunsundar.pharmacy@sathyabama.ac.in ORCID iD: https://orcid.org/0000-0002-2483-7679

## Data availability statement

The data in this research article is not sensitive in nature and is accessible in the public domain. The data is therefore available and not of a confidential nature.

## Provenance and peer review

Not commissioned, externally peer-reviewed.
